# The distribution of reproductive risk factors disclosed the heterogeneity of receptor-defined breast cancer subtypes among Tanzanian women

**DOI:** 10.1186/s12905-021-01536-6

**Published:** 2021-12-20

**Authors:** Linus P. Rweyemamu, Gokce Akan, Ismael C. Adolf, Erick P. Magorosa, Innocent J. Mosha, Nazima Dharsee, Lucy A. Namkinga, Sylvester L. Lyantagaye, Abdolrahman S. Nateri, Fatmahan Atalar

**Affiliations:** 1 Department of Molecular Biology and Biotechnology, University of Dar es Salaam, P.O Box 35179, Dar es Salaam, Tanzania; 2 Mbeya College of Health and Allied Sciences, University of Dar es Salaam, P.O Box 608, Mbeya, Tanzania; 3 MUHAS Genetic Laboratory, Department of Biochemistry, Muhimbili University of Health and Allied Sciences, P.O Box 65001, Dar es Salaam, Tanzania; 4 Department of Anatomical Pathology, Muhimbili National Hospital, P.O Box 65000, Dar es Salaam, Tanzania; 5 Academic, Research and Consultancy Unit, Ocean Road Cancer Institute, P.O Box 3592, Dar es Salaam, Tanzania; 6 Cancer Genetics and Stem Cell Group, Division of Cancer and Stem Cells, School of Medicine, BioDiscovery Institute, University of Nottingham, Nottingham, NG7 2UH UK; 7 Department of Rare Diseases, Child Health Institute, Istanbul University, Istanbul, 34093 Turkey

**Keywords:** Breast cancer, Reproductive risk factors, Molecular subtype, Heterogeneity, Tanzania

## Abstract

**Background:**

Recent epidemiological studies suggest that reproductive factors are associated with breast cancer (BC) molecular subtypes. However, these associations have not been thoroughly studied in the African populations. The present study aimed to investigate the prevalence of BC molecular subtypes and assess their association with reproductive factors in Tanzanian BC patients.

**Methods:**

This hospital-based case-only cross-sectional study consisted of 263 histologically confirmed BC patients in Tanzania. Clinico-pathological data, socio-demographic characteristics, anthropometric measurements, and reproductive risk factors were examined using the Chi-square test and one-way ANOVA. The association among reproductive factors and BC molecular subtypes was analyzed using multinomial logistic regression. The heterogeneity of the associations was assessed using the Wald test.

**Results:**

We found evident subtype heterogeneity for reproductive factors. We observed that post-menopausal status was more prevalent in luminal-A subtype, while compared to luminal-A subtype, luminal-B and HER-2 enriched subtypes were less likely to be found in post-menopausal women (OR: 0.21, 95%CI 0.10–0.41, *p* = 0.001; OR: 0.39, 95%CI 0.17–0.89, *p* = 0.026, respectively). Also, the luminal-B subtype was more likely to be diagnosed in patients aged ≤ 40 years than the luminal-A subtype (OR: 2.80, 95%CI 1.46–5.32, *p* = 0.002). Women who had their first full-term pregnancy at < 30 years were more likely to be of luminal-B (OR: 2.71, 95%CI 1.18–4.17, *p* = 0.018), and triple-negative (OR: 2.28, 95%CI 1.02–4.07, *p* = 0.044) subtypes relative to luminal-A subtype. Furthermore, we observed that breastfeeding might have reduced odds of developing luminal-A, luminal-B and triple-negative subtypes. Women who never breastfed were more likely to be diagnosed with luminal-B and triple-negative subtypes when compared to luminal-A subtype (OR: 0.46, 95%CI 0.22–0.95, *p* = 0.035; OR: 0.41, 95%CI 0.20–0.85, *p* = 0.017, respectively).

.

**Conclusion:**

Our results are the first data reporting reproductive factors heterogeneity among BC molecular subtypes in Tanzania. Our findings suggest that breast-feeding may reduce the likelihood of developing luminal-A, luminal-B, and triple-negative subtypes. Meanwhile, the first full-term pregnancy after 30 years of age could increase the chance of developing luminal-A subtype, a highly prevalent subtype in Tanzania. More interventions to promote modifiable risk factors across multiple levels may most successfully reduce BC incidence in Africa.

**Supplementary Information:**

The online version contains supplementary material available at 10.1186/s12905-021-01536-6.

## Introduction

Breast cancer (BC) is the most prevalent malignancy and the leading cause of cancer deaths globally. BC accounts for about 11.6% of all cancers worldwide, and reports showed that in 2018, 2.1 million new BC cases were diagnosed, of which 627,000 individuals lost their lives [1]. BC incidences are higher in developed countries than in developing countries, including sub-Saharan Africa (SSA), though mortalities are disproportionately higher in the latter [2]. In Tanzania, BC is the second most leading cause of mortality among women after cervical cancer [3, 4]. The age-standardized BC incidence is 19.4 new cases per 100,000 women, and the age-standardized mortality is 9.7 per 100,000 women, meaning that 50% of women diagnosed with BC in the country die of the disease. It is reported that about 80% of women diagnosed with BC in Tanzania present with a late-stage (stage III and IV) disease when the treatment is less effective and most patients cannot afford the treatment-associated costs [5].

BC is an umbrella term used to describe heterogeneous diseases diverse in morphology, pathology, histology, and molecular aspects [6]. The traditional BC classification depended on the histopathologic characteristics that encompass the tumor size, the nodal status, the local invasion, and the distant metastasis, which ultimately dictated the treatment modalities [7]. However, since the last two decades, BC classification has been overturned from pathologic types to molecular subtypes determined by gene expression profiling [8]. Gene expression profiling of BC is not yet fully available in many clinical settings due to its high costs and extensive resources needed [9]. Thus, the surrogate definition of BC molecular subtypes is based on immunohistochemistry (IHC) expression of three main protein markers; the estrogen receptor (ER), the progesterone receptor (PR), and the overexpression of human epidermal growth factor receptor-2 (HER-2/neu). Additional markers such as proliferation index Ki-67 and basal cytokeratin 5/6 (CK5/6) are crucial in BC molecular subtyping, though not well adopted in most oncology diagnostic centers, particularly in Sub-Saharan Africa (SSA) [10, 11].

In many clinical settings, the standard BC management modalities mainly depend on the tumor molecular subtype that has changed the paradigm of BC treatment [8]. Based on ER, PR, and HER-2 key markers, four major BC molecular subtypes exist; Luminal-A, Luminal-B, HER-2 enriched, and Triple-negative/basal-like. These present subtypes of differences in incidence, prognosis, recurrence, response to the treatment regime, preference in metastatic organ, and survival outcome [7]. The risk factors for BC are well reviewed in the literature. Socio-demographic factors such as age at BC diagnosis, smoke exposure, alcohol consumption, body mass index (BMI) (obesity or overweight), family history of BC, and reproductive risk factors such as early menarche, late menopause, parity, age at first full-term pregnancy, breast-feeding, duration of breast-feeding and use of hormone replacement therapy (HRT) are associated with BC [12, 13].

Recent studies suggested that reproductive risk factors impact tumor subtypes of BC and BC etiology in women of different ethnic backgrounds worldwide [14]. Several studies have indicated that these reproductive risk factors are related to ER/PR receptors positive BC tumor subtypes [15–17]. A study in Northern China revealed that women aged 40 years or below who breast-fed for at least 12 months had a reduced risk of luminal-B and triple-negative subtypes while parity had a strong protection against luminal-A and luminal-B subtypes in both young and older women [14]. Indeed, parous women were more likely to be diagnosed with triple-negative subtype regardless of the age at BC diagnosis [18]. Also, a recently published study reported that the age of first pregnancy was significantly associated with the luminal-A subtype [19].

Nevertheless, the underlying biological mechanisms remain poorly understood and are still under investigation. Moreover, most of the available data regarding the BC molecular subtypes, their distribution and association with reproductive risk factors were derived from studies in Europe, America and Asia. Such studies are very limited in Africa, particularly in the East African population. Thus, the present study's objective was to establish the molecular subtype profiles of BC patients and then assess the association between the reproductive risk factors and these BC molecular subtypes.

## Methods

### Study design and setting

The present case-only hospital-based cross-sectional study was carried out at Ocean Road Cancer Institute (ORCI) between September 2019 and June 2020 in Tanzania. Tanzania is a lower-middle-income country in Eastern Africa with an estimated population of about 59 million in 2020. The country spans about 5° south of the Equator, occupying an area of 945,087-square kilometers. ORCI is the only national specialized cancer facility located at the shores of the Indian Ocean in Dar es Salaam city, Tanzania. The facility was established back during German colonial times in 1895. It was declared an independent, autonomous institute by an Act of Parliament later in 1996. The ORCI, among other core services, provides the primary BC screening and treatment and receives referral cases from Muhimbili National Hospital (MNH) and other private and public hospitals for Radiotherapy (including Brachytherapy), Chemotherapy, Hormonal/Endocrine therapy, Immunotherapy, and palliative care. The facility serves both local and foreign clients in which about 28,000 cancer confirmed patients, 10,000 cancer screening patients and 12,000 non-cancer patients are attended annually.

### Study population

The study participants reported here were of Tanzanian origin/ethnicity, and confirmed the BC diagnosis with the histologic examination between June 2010 and January 2020. Patients of other ethnicities apart from Tanzanian, patients missing important clinico-pathological data in the file, and patients having a score of 2+ for HER-2 expression were excluded. A total of 263 patients constituted the subset for this study. The participants recruited prospectively had their samples obtained as core biopsies following the hospital standard protocol. Ethical clearances with reference numbers *10/Vol/XX/16* and *NIMR/HQ/R.8a/Vol. IX/3255* were issued by the host institute (ORCI) and Tanzania National Institute for Medical Research (NIMR), respectively. All methods were also performed following the relevant guidelines and regulations (Declaration of Helsinki). Written informed consent from each study participant was obtained.

### Data collection

Participants were asked about their information as follows; socio-demographic characteristics including age, marital status (married, single and widowed), occupation (peasant, housewife, business and employed), place of origin, smoking during adolescence and adulthood (yes, no), alcohol during adolescence and adulthood (yes, no), anthropometric measurements (including height and weight) for BMI calculation (defined as < 25, 25.0–30 and > 30.0 kg/m^2^), family history of BC (yes, no), and reproductive factors including menarche age (< 12, 13–14, ≥ 15 years), parity (nulliparous, 1–2 and > 3 children), age at first full-term pregnancy (< 30 and ≥ 30 years), breast-feeding (yes, no), breast-feeding duration (never, > 15 and ≥ 15), menopausal status (pre-menopausal, post-menopausal), HRT use (yes, no), oral contraceptive use (yes, no) and duration of oral contraceptive use (< 48 and ≥ 48 months) during face-to-face interviews by the well-trained and experienced nurses. *Parity* was defined as having one or more children. First, *full-term pregnancy* was defined as the first pregnancy that was completed at least 39 weeks. *Post-menopausal* status was defined as the cessation of menstrual cycles within the past 12 months prior to the interview. *Pre-menopausal* status was defined as regular menstrual cycles at 12 months. A physician extracted clinico-pathological data for all participants from their hospital electronic and/or physical files. Characteristics including metastasis status, status for tumor node metastasis (TNM) staging, tumor histological type, laterality, and age at BC diagnosis and treatment modalities at ORCI were abstracted.

### Molecular subtypes characterization

The study material consisted of core needle or surgical samples fixed in 10% formalin. The classical histology techniques using hematoxylin and eosin staining were carried on formalin-fixed paraffin-embedded (FFPE) breast tissue blocks. The malignant tumors were classified according to the World Health Organization (WHO) classification of breast tumors [20] and graded with the criteria of Elston and Ellis [21]. The FFPE tissue sections were studied in the MNH pathology laboratory for histological analysis and IHC. The minority patients had undergone such examinations at either Kilimanjaro Christian Medical Center (KCMC), located in Kilimanjaro or Bugando Medical Center (BMC), located in Mwanza. The ER, PR, and HER2/neu status were determined by immunohistochemistry upon the FFPE blocks of breast carcinoma patients. Both ER and PR were scored based on an Allred scoring system that considers the percentage of stained cells (scale of 0–5) and the staining intensity (scale of 0–3). A minimum of 1% stained cells was considered for ER/PR positive tumors. An aggregate (from both percentages of stained cells and intensity) score of 3 or more was considered positive for ER and PR markers. HER-2 marker was scored on a scale of 0–3+. A score of 0 or 1+ was regarded as HER-2 negative, a score of 2+ was regarded as equivocal (excluded in this study).

In contrast, a score of 3+ was regarded as HER-2 positive as per recommendations from the College of American Pathologists/American Society of Clinical Oncology (CAP/ASCO) [22]. The pathological stage was established based on tumor status, lymph node involvement status, and metastasis status as per the TNM staging system [23]. We classified BC cases into four major molecular subtypes based on IHC expression of ER/PR/HER-2 markers as shown below:

- Luminal-A (ER+/PR+/HER-2− or ER+/PR−/HER-2− or ER−/PR+/HER-2−),
- Luminal-B (ER+/PR+/HER-2+ or ER+/PR−/HER-2+ or ER−/PR+/HER-2+),
- HER-2-enriched (ER−/PR−/HER-2+) and
- Triple-negative (ER−/PR−/HER-2−).

### Statistical analyses

All data collected were organized in an excel database for windows 10 (Microsoft Corporation, Redmond, WA, USA) and analyzed in Statistical Package for Social Sciences 25.0 (IBM SPSS, Inc., Chicago, IL, USA). Differences between BC molecular subtypes about clinicopathologic, anthropometric, sociodemographic and reproductive characteristics were examined using One-Way ANOVA for the quantitative variables and Chi-square (χ^2^) tests for the categorical variables. The results were expressed as the mean ± standard deviation, or percentage, wherever appropriate. Relative risks were assessed in BC molecular subtypes by calculating odds ratios (ORs) and 95% confidence intervals (CIs) that were considered separate outcomes. Their respective risks were modelled via multinomial logistic regression. Heterogeneity was formally assessed with the Wald test, testing the null hypothesis that the risk associated with BC was the same across all molecular subtypes.

Additionally, multinomial logistic regression was performed using luminal-A cases as a reference group. The combined effect of parity and breast-feeding was also assessed on the risk of BC in all molecular subtypes using multivariable logistic regression. Analysis of covariance (ANCOVA) was used to determine possible confounders. We considered the following factors as potential confounders in all multivariable models: Age, BMI, and family history of BC (yes/no). Statistical significance was considered when *p* < 0.05.

## Results

A total of 263 BC patients with a mean age of 47.99 ± 11.61 years formed the study population. Most of the study participants were from urban areas (59.3%). The most significant proportion of the present study cohort originated from the Northern zone of Tanzania (33.8%). Table 1 summarizes the frequency of clinico-pathological, anthropometric, sociodemographic characteristics and reproductive risk factors of the study population. Invasive ductal carcinoma of no specific type (IDC-NST) was the most prevalent histological tumor type in this study, accounting for 88.2%. TNM pathologic stage III carcinomas were the most common, accounting for 55.5%, 27.4% stage IV, 15.6% stage II and 1.5% stage I.

**Table 1:** Clinico-pathological, anthropometric and socio-demographic characteristics parameters of study group

Characteristics	Number of patients, n (%)
*Current age (years) mean* ± *SD*	47.99 ± 11.61
*Age at diagnosis (years) mean* ± *SD*	44.49 ± 10.81
*Histological type*	
IDC-NST	232 (88.2)
ILC	13 (4.9)
MC	5 (1.9)
Others	13 (4.9)
*TNM pathological stage*	
I	4 (1.5)
II	41 (15.6)
III	146 (55.5)
IV	72 (27.4)
*Tumor laterality*	
Left	127 (48.3)
Right	127 (48.3)
Bilateral	9 (3.4)
*ER status*	
Positive (+)	169 (64.3)
Negative (−)	94 (35.7)
*PR status*	
Positive (+)	139 (52.9)
Negative (−)	124 (47.1)
*HER2 status*	
Positive ( +)	94 (35.7)
Negative (−)	169 (64.3)
*Molecular subtype*	
Luminal-A	117 (44.5)
Luminal-B	59 (22.4)
HER-2 enriched	29 (11.0)
Triple-negative	58 (22.1)
*Menopausal status*	
Pre-menopause	119 (45.2)
Post-menopause	144 (54.8)
< 50 years old	91 (63.2)
≥ 50 years old	53 (36.8)
*Family history of BC*	
Yes	55 (20.9)
No	208 (79.1)
*BMI*	
Normal weight < 25 kg/m^2^	100 (38)
Overweight 25–30 kg/m^2^	72 (27.4)
Obese > 30 kg/m^2^	91 (34.6)
*Menarche*	
Early < 12 years old	11 (4.2)
Normal 13–14 years old	115 (43.7)
Late > 15 years old	137 (52.1)
*Age at first full-term pregnancy*	
< 30 years old	198 (80.5)
≥ 30 years old	48 (19.5)
*Parity*	
Nulliparous	27 (10.3)
1–2 Children	88 (33.5)
≥ 3 Children	148 (56.3)
*Breast-feeding*	
Breast-feeding duration (months) mean ± SD	15.5 ± 6.5
Never	63 (24)
Yes	200 (76)
< 15 months	48 (18.3)
≥ 15 months	152 (57.8)
*Contraceptive use*	
Yes	113 (43)
< 48 months	34 (30.1)
≥ 48 months	79 (69.9)
No	150 (57)
*HRT use*	
Yes	0 (0)
No	263 (100)
*Smoke exposure*	
Yes	4 (1.5)
No	259 (98.5)
*Alcohol consumption*	
Yes	48 (18.3)
No	215 (81.7)
*Residance*	
Rural	107 (40.7)
Urban	156 (59.3)
*Place of origin*	
Central zone	37 (14.1)
Western zone	14 (5.3)
Eastern zone	50 (19)
Northern zone	89 (33.8)
Southern zone	55 (20.9)
Lake zone	18 (6.8)
*Occupation*	
Peasant	107 (40.7)
Housewife/dependant	70 (26.6)
Business/entrepreneur	63 (24)
Employed	16 (6.1)
Others	7 (2.6)
*Marital status*	
Married	179 (68.1)
Single	25 (9.5)
Widowed/separated/divorced	59 (22.4)

*TNM* tumor node metastasis, *IDC-NST* ınvasive ductal carcinoma of no specific type, *ILC* ınvasive lobular carcinoma, *MC* mucinous carcinoma, *ER* estrogen receptor, *PR* progesterone receptor, *HER2* Human epidermal growth factor receptor 2, *BMI* body mass index, *HRT* hormone replacement therapy, *SD* Standard deviation

**Fig. 1:**
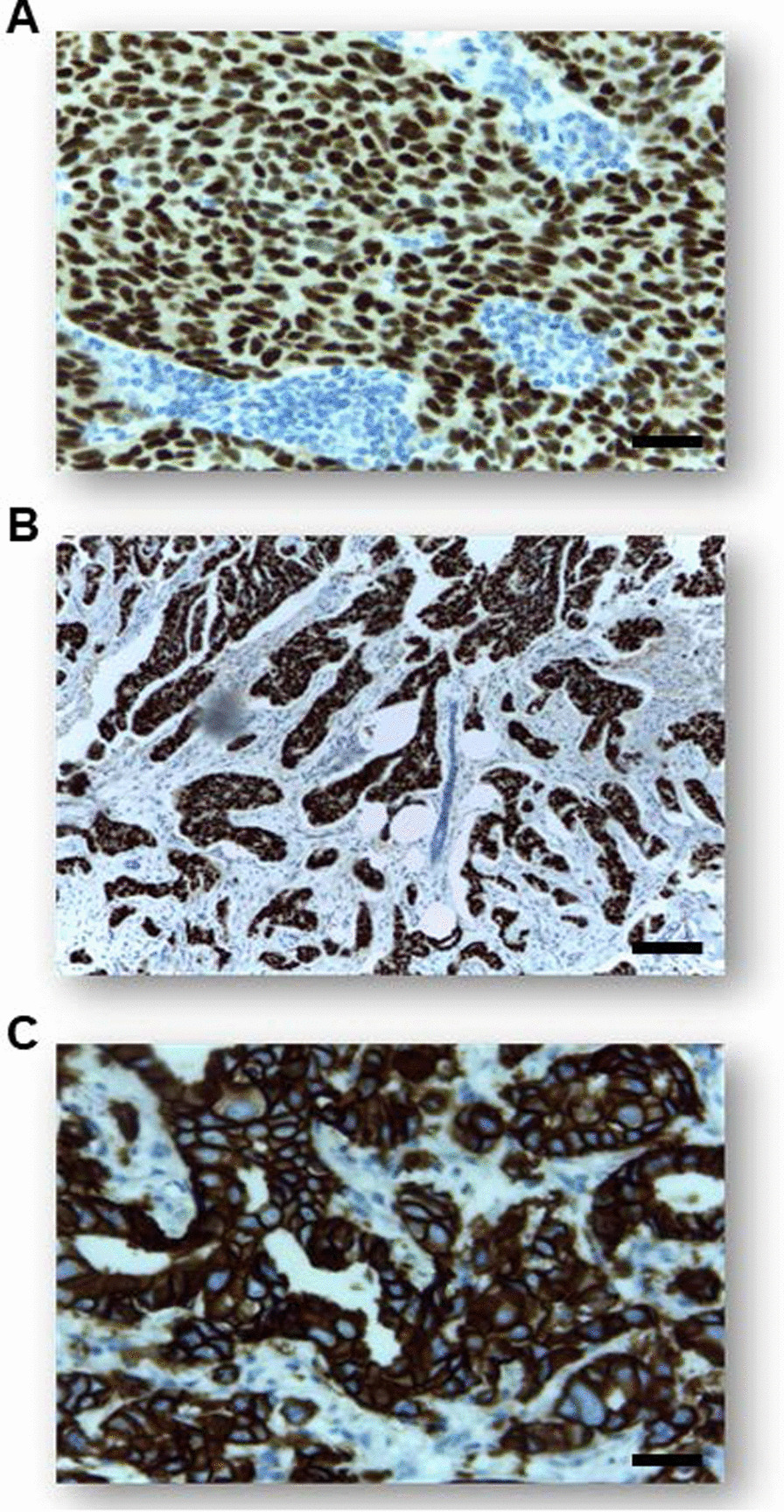
Examples of well-differentiated BC biomarkers by immunohistochemistry staining. **a** Estrogen receptor-positive staining, Scale bar: 50 µm **b** Progesterone receptor-positive staining, Scale bar: 65 µm **c** HER2 positive staining, Scale bar: 50 µm

**Fig. 2:**
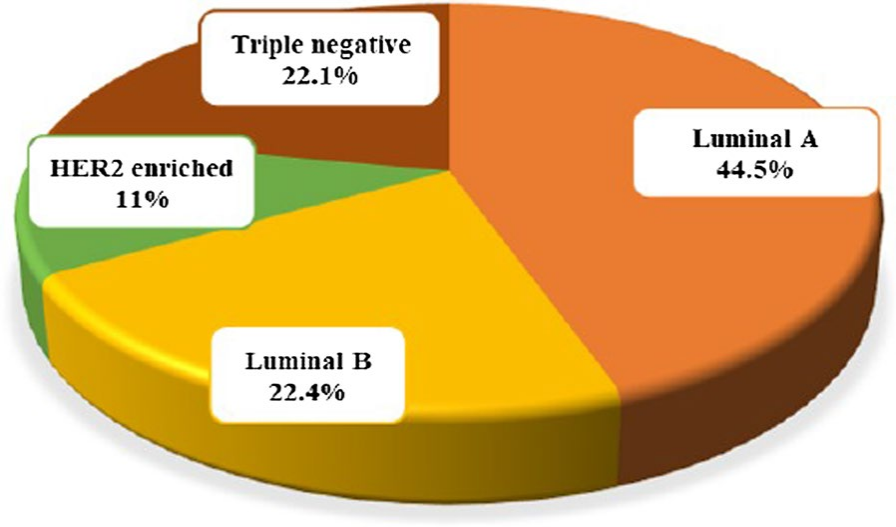
Distribution of cases as per molecular subtypes. The values were calculated using the Chi-square test, and the data were expressed in percentages. The figure was generated using GraphPad Prism version 8.0 (GraphPad Software Inc., San Diego, CA, USA)

The IHC data revealed that among 263 patients, 65.5% expressed hormone receptor-positive (ER+ and/or PR+), 64.3% expressed estrogen receptor-positive (ER+), 52.9% expressed progesterone receptor-positive (PR+) and the proportion of patients overexpressing HER2 (HER2+) was 35.7% (Table 1). The largest proportions of cases were classified as luminal-A (44.5%) followed by luminal-B (22.4%), triple-negative (22.1%), and HER-2 enriched (11%) subtype (Figs. 1 and 2).

BC was the most common in post-menopausal women (54.8%), and within this group, the majority (63.2%) had attained menopause before 50 years of age. Additionally, the majority (52.1%) of the study group had late menarche (i.e. after 15 years old). Most BC patients in the present study had at least three children (56.3%). The majority (80.5%) had their first full-term pregnancy before 30 years of age, and 57.8% of the study cohort reported at least 15 cumulative months of breast-feeding. The 43% of BC patients had a history of contraceptive use with a mean duration of use of 69.64 months. With BMI (kg/m^2^), nearly 62% of the women were either overweight (27.4%) or obese (34.6%). Almost all (98.5%) study participants were not exposed to smoking, and only 18.3% had used alcohol. The majority (79.1%) of patients reported no history of BC in their families.

Table 2 shows the distribution of clinico-pathological parameters and reproductive risk factors in each subtype. The molecular subtypes differed significantly by histological type (*p* = 0.012). The triple-negative subtype showed the highest prevalence of invasive lobular carcinoma (ILC) histological type compared to other subtypes, 15.5% versus 2.6% luminal-A, 0% luminal-B and 3.4% HER-2 enriched. When we analyzed the reproductive risk factors in each subtype, we observed no heterogeneity in terms of menarche and family history of BC parameters among the four subtypes, as the differences of distributions were not statistically significant. Meanwhile, the subtypes differed significantly by age at diagnosis (*p* = 0.001). The Luminal-A subtype was more prevalent at older ages (mean = 48), and the luminal-B subtype was more prevalent at younger ages (mean = 39). Therefore, we assessed the possible interaction between reproductive factors and molecular subtypes using chi-square (χ^2^) tests (Additional file 1: Table S1). To investigate the association between age at BC diagnosis and molecular subtypes, we divided breast cancer patients into two subgroups according to the age at BC diagnosis (< 40 years old and ≥ 40 years old). Compared to other subtypes (44.2%), the luminal-A subtype (62.1%) was found to be more likely diagnosed at the age of ≥ 40 years (OR: 2.06, 95%CI 1.14–3.70, *p* = 0.014).

**Table 2:** Characteristics and reproductive factors of BC patients by molecular subtypes

Characteristics	Luminal-A (n = 117)	Luminal-B (n = 59)	HER-2 enriched (n = 29)	Triple-negative (n = 58)	*p* value
*Age at diagnosis (years)*^*a*^	48.78 ± 12.19	39.73 ± 6.40	41.76 ± 9.14	44.49 ± 10.81	**0.001**
*Histological type, n (%)*^*b*^					
IDC-NST	105 (89.7)	55 (93.2)	26 (89.7)	46 (79.3)	**0.012**
ILC	3 (2.6)	0 (0)	1 (3.4)	9 (15.5)	
MC	2 (1.7)	2 (3.4)	1 (3.4)	0 (0)	
Others	7 (6)	2 (3.4)	1 (3.4)	3 (5.2)	
*TNM pathological stage, n (%)*^*b*^					
I	1 (0.9)	0 (0)	2 (6.9)	1 (1.7)	0.059
II	24 (20.5)	4 (6.8)	2 (6.9)	11 (19)	
III	64 (54.7)	33 (55.9)	15 (51.7)	34 (58.6)	
IV	28 (23.9)	22 (37.3)	10 (34.5)	12 (20.7)	
*Tumor laterality, n (%)*^*b*^					
Left	60 (51.3)	31 (52.5)	11(37.9)	25 (43.1)	0.377
Right	53 (45.3)	27 (45.8)	18 (62.1)	29 (50)	
Bilateral	4 (3.4)	1 (1.7)	0 (0)	4 (6.9)	
*Menopausal status, n (%)*^*b*^					
Pre-menopause	38 (32.5)	41 (69.5)	16 (55.2)	24 (39.1)	**0.001**
Post-menopause	79 (67.5)	18 (30.5)	13 (44.8)	34 (60.9)	
*Family history of BC, n (%)*^*b*^					
Yes	25 (21.4)	12 (20.3)	4 (13.8)	14 (24.1)	0.734
No	92 (78.6)	47 (79.7)	25 (86.2)	44 (75.9)	
*Menarche, n (%)*^*b*^					
Early < 12 years old	5 (4.3)	3 (5.1)	2 (6.9)	1 (1.7)	0.802
Normal 13–14 years old	54 (46.1)	27 (45.7)	10 (34.5)	24 (41.4)	
Late > 15 years old	58 (49.6)	9 (49.2)	17 (58.6)	33 (56.9)	
*Age at first full-term pregnancy, n (%)*^*b*^					
< 30 years old	75 (74.8)	46 (86.8)	21 (79.3)	43 (87.8)	0.145
≥ 30 years old	25 (25.2)	9 (13.2)	6 (20.7)	10 (12.2)	
*Parity, n (%)*^*b*^					
Nulliparous	17 (13.7)	4 (6.8)	2 (6.9)	5 (10.3)	0.103
1–2 Children	35 (30.8)	26 (44.1)	7 (24.1)	20 (33.8)	
≥ 3 Children	65 (55.6)	29 (49.2)	20 (69)	33 (55.9)	
*Breast-feeding, n (%)*^*b*^					
Yes	96 (82.1)	40 (67.8)	22 (75.9)	38 (65.5)	0.058
No	21 (17.9)	19 (32.2)	7 (24.1)	20 (25.5)	
*Breast-feeding duration, n (%)*^*b*^					
< 15 months	19 (19.8)	10 (25)	6 (27.3)	12 (31.6)	0.516
≥ 15 months	77 (80.2)	30 (75)	16 (72.7)	26 (68.4)	

The *p*-values less than 0.05 (typically ≤ 0.05) as being statistically significant and were bolded

*TNM* tumor node metastasis, *HER2* human epidermal growth factor receptor-2, *IDC-NST* invasive ductal carcinoma of no specific type, *ILC* invasive lobular carcinoma, *MC* mucinous carcinoma

^a^The values are calculated using one-way ANOVA and the data are given mean ± standard deviation

^b^The values are calculated using Chi-square test and the data are expressed in percentages. In all cases, differences were considered significant at *p* < 0.05

Molecular subtypes also showed a very significant difference according to menopausal status (*p* = 0.001). Indeed, luminal-A and triple-negative subtypes (67.5% and 60.9%, respectively) were post-menopausal, while luminal-B and HER-2 enriched subtypes (69.5% and 55.2%, respectively) were pre-menopausal. When we analyzed menopausal status in all subtypes separately, the luminal-A subtype showed a higher likelihood of being post-menopausal than other non-luminal-A (44.2%) subtypes (OR: 2.69, 95%CI 1.16–4.47, *p* = 0.001). Meanwhile, the luminal-B subtype was more likely to be pre-menopausal than the non-luminal-B (38.2%) subtypes (OR: 3.89, 95%CI 2.07–7.33, *p* = 0.001).

Furthermore, there was heterogeneity in terms of parity, breast-feeding, and breast-feeding duration among subtypes. Most of the luminal-A cases had ≥ 1 child and breast-fed for ≥ 15 months compared to other subtypes, but the differences were not statistically significant. We divided the BC patients into two subgroups to investigate the possible interaction of parity and luminal-A subtype (Nulliparous and ≥ 1 Child). We observed that nulliparous women was more likely to have luminal-A subtype (14.7%) than non-luminal-A (5.4%) cases (OR: 2.15, 95%CI 0.93–4.94, *p* = 0.035). In addition, women who had their first full-term pregnancy at ≥ 30 years old had a more likelihood of having luminal-A subtype than non-luminal-A (14.5%) subtypes (OR: 0.50, 95%CI 0.26–0.95, *p* = 0.004). A majority of the patients (82.1%) with luminal-A subtype had also breast-fed their babies. When compared luminal-A subtypes (17.9%) to non-luminal-A subtypes (31.3%), we noted that women who breast-fed were less likely to be of luminal-A subtype (OR: 2.1, 95%CI 1.17–3.78, *p* = 0.009).

Further, we analyzed the combined effect of parity and breast-feeding as a risk factor of BC in all subtypes. We divided BC patients into three subgroups; the first group was women with ≥ 1full-term pregnancy who breast-fed for at least 15 months. The second group was those with ≥ 1full-term pregnancy who never breast-fed, and the third group was nulliparous women. Our findings revealed that women with ≥ 1full-term pregnancy who breast-fed at least 15 months were less likely to develop luminal-A subtype than non-luminal-A cases. However, this difference was not statistically significant (OR: 1.09, 95%CI 0.25–1.25, *p* = 0.506) (data not shown).

Subsequently, we assessed associations between reproductive factors and subtypes by multinomial logistic regression analysis, and the results are shown in Table 3. We observed that women aged 40 years or younger were more likely to develop the luminal-B subtype than the luminal-A subtype (OR: 2.80, 95%CI 1.46–5.32, *p* = 0.002). Moreover, when women having first full-term pregnancy at or after 30 years of age were taken as a reference group, we noted that women having the first full-term pregnancy before 30 years old were more likely to being luminal-B (86.3%) and triple-negative (87.8%) subtypes when compared to luminal-A subtype (OR: 2.71, 95%CI 1.18–4.17, *p* = 0.018; OR: 2.28, 95%CI 1.02–4.07, *p* = 0.044, respectively). Similarly, when breast-fed women were taken as references group; the women who never breast-fed were more likely to develop luminal-B (32.2%) and triple-negative (25.5%) subtypes when compared to luminal-A subtype (OR: 0.46, 95%CI 0.22–0.95, *p* = 0.035; OR: 0.41, 95%CI 0.20–0.85, *p* = 0.017, respectively). ​

**Table 3:** The odds ratios and 95% confidence intervals of reproductive risk factors by BC subtypes

Risk factor	Luminal-A	Luminal-B	HER-2 enriched	Triple-negative
	OR (95% CI) *p* value	OR (95% CI) *p* value	OR (95% CI) *p* value	OR (95%CI) *p* value
*Age at diagnosis*				
< 40 years old	1.00 (Ref)	2.80 (1.46–5.32) **0.002**	2.04 (1.89–4.64) 0.089	1.66 (0.88–3.13) 0.119
≥ 40 years old (Ref)				
*Family history of BC*				
Yes	1.00 (Ref)	0.94 (0.43–2.03) 0.940	0.59 (0.18–1.85) 0.364	1.17 (0.55–2.47) 0.353
No (Ref)				
*Menarche*				
Early < 12 years	1.00 (Ref)	1.20 (0.26–5.73) 0.812	1.36 (0.24–1.67) 0.724	0.35 (0.29–1.33) 0.352
Normal 13–14 years		1.19 (0.52–1.90) 0.892	0.63 (0.26–1.50) 0.298	0.81 (0.41–1.48) 0.781
Late > 15 years (Ref)				
*Menopausal status*				
Pre-menopause	1.00 (Ref)	0.21 (0.10–0.41) **0.001**	0.39 (0.17–0.89) **0.026**	0.68 (0.35–1.30) 0.681
Post-menopause (Ref)				
*Age at first full-term pregnancy*				
< 30 years old	1.00 (Ref)	2.71 (1.18–4.17) **0.018**	1.85 (0.68–2.51) 0.223	2.28 (1.02–4.07) **0.044**
≥ 30 years old (Ref)				
*Parity*				
Nulliparous (Ref)	1.00 (Ref)	0.42 (0.13–1.33) 0.428	0.44 (0.15–1.95) 0.286	0.43 (0.14–1.36) 0.153
≥ 1 Child				
*Breastfeeding*				
Ever(Ref)	1.00 (Ref)	0.46 (0.22–0.95) **0.035**	0.69 (0.26–1.81) 0.450	0.41 (0.20–0.85) **0.017**
Never				

The *p*-values less than 0.05 (typically ≤ 0.05) as being statistically significant and were bolded

The ORs and 95%CIs were found by multinomial logistic regression comparing luminal-A cases as the reference group. All odds ratios (OR) are adjusted age, BMI and family history of BC (yes, no). In all cases, differences were considered significant at *p* < 0.05

*CI* confidence interval, *OR* odds ratio, *HER2* human epidermal growth factor receptor 2, *Ref* reference

The effect of menopausal status was heterogeneous across the subtypes (p_heterogeneity_ = 0.001). Post-menopause women as a reference group, pre-menopausal status was more prevalent in luminal-B (69.5%) and HER-2 enriched (55.2%) subtypes. Post-menopause women were less likely to develop luminal-B, and HER-2 enriched subtypes compared to luminal-A subtype (OR: 0.21, 95%CI 0.10–0.41, *p* = 0.001; OR: 0.39, 95%CI 0.17–0.89, *p* = 0.026, respectively). In contrast, there was no statistical evidence of heterogeneity for family history of BC, menarche, and parity among BC subtypes (p_heterogeneity_ > 0.05).

## Discussion

In this case-only hospital-based cross-sectional study, we observed that majority of the BC patients enrolled came from urban residences rather than rural residences. We observed that a substantial number of BC patients originated from the Northern part of Tanzania compared to the other zones. We speculated that the women have limited reach to diagnostic facilities in rural residences and possibly die with BC undiagnosed. Similar speculations were presented from different regions of Africa [24].

The IHC evaluation among 263 BC patients resulted in nearly 70% of BC patients expressing ER and/or PR receptors markers; hence can benefit from hormonal therapy. We also observed that luminal-A comprises the most common subtypes among the studied population (44.5%), and this was followed by luminal-B (22.4%), triple-negative (22.1%) and HER-2 enriched (11%) (Fig. 2). A similar BC molecular subtypes prevalence pattern was reported in Eastern Africa country, Ethiopia [10] and Northern Africa country, Morocco [25] whereby luminal-A was the commonest subtype and HER-2 enriched was the rarest subtype. Other studies from Asian and European countries have also reported the highest prevalence of luminal-A subtype in their regions [26, 27]. Our findings differ from the Tanzanian report that showed nearly half (45.6%) of cases previously analyzed for ER, PR and HER-2 receptor status were triple-negative [28]. The difference might be attributed to their small sample size being only 53 BC samples analyzed for hormonal receptors, thereby limiting comparability with our findings.

However, our findings differ from many African studies, as summarized in Additional file 1: Table S2. Most African studies show that triple-negative ranks the first or the second most prevalent subtype among women diagnosed with BC [29]. Triple-negative tumors are aggressive, younger age at diagnosis, and are most frequently seen in BC patients of African ancestry [30]. The relatively low prevalence of triple-negative subtypes observed in this study could be due to the study design. The data presented here are of BC patients diagnosed with the disease between 2010 and 2020 and who consented to participate. There might be a significant number of triple-negative BC patients who died during treatment, considering that triple-negative tumors are aggressive and patients have shorter survival compared to other subtypes [30]. Indeed, African data on the distribution of molecular subtypes are inconsistent, perhaps due to many factors, including study design and relatively small sample-sized studies. Triple-negative subtype might be overestimated in many African settings due to the lack of adequate state of the art facilities needed to fix paraffin-embedded tissues and antibodies staining rendering many tumors negative for ER, PR and HER-2 markers.

Histopathological analysis showed that IDC-NST was the most prevalent histologic type in this study cohort ranging from 78.3 to 93.2% in all four BC subtypes. This prevalence is consistent with the findings from other published studies in various African and Asian countries [31, 32]. The ILC was the second most frequent and was significantly abundant in triple-negative subtypes (75.2%, *p* = 0.014) in our study, which partly matches the findings from the retrospective study in the Kingdom of Saudi Arabia [33], and conflicts with the findings from Northern Africa that showed ILC was more abundant in luminal-A subtypes [34].

We further analyzed the association of reproductive risk factors and BC molecular subtypes. Reproductive risk factors were differentially associated with molecular subtypes of BC in the present study. We observed a clear BC molecular subtypes heterogeneity for reproductive factors. The most prominent reproductive factors were age at BC diagnosis, menopausal status, parity, age at the first full-term pregnancy, and breast-feeding in different age groups. The BC molecular subtypes differed significantly by age at BC diagnosis. The luminal-B subtype was more prevalent at younger ages (mean = 39), and the luminal-A subtype was more prevalent at older ages (mean = 48). Women 40 years of age or older had a more likelihood of being diagnosed with luminal-A subtype. Hence, 40+ years of age is a likely risk factor for the luminal-A subtype in our study cohort. Previous studies have examined the association of age at BC diagnosis and molecular subtypes. A study in Turkish women demonstrated that advanced age (40+ years) was a risk factor for both luminal-A and triple-negative subtypes [35].

On the other hand, studies in Western countries and the USA show that the triple-negative subtype is more likely to be diagnosed at a younger age [30]. This was not observed in this study as the mean age at BC diagnosis for triple-negative and HER-2 enriched subtypes were almost the same (Table 2). In addition, the analysis of multinomial logistic regression showed that the luminal-B subtype was more likely to be diagnosed among women aged 40 years or younger than the luminal-A subtype (Table 3).

In this study, hormonal receptor-positive subtypes (luminal-A and luminal-B) were differential in menopausal status. Women who had attained menopause (postmenopausal) were likely to be of luminal-A subtype, whereas pre-menopausal women were determined to be of luminal-B subtype. Additionally, our multinomial logistic regression analysis supported these results. A prior study by Turkoz et al. reported that postmenopausal women who used HRT for more than 5 years had increased odds of developing luminal-A subtype [35]. However, in our study, none of the study participants reported using HRT.

Parity was another related reproductive risk factor of BC in our study population. Our findings showed that the luminal-A subtype was more likely to be diagnosed in nulliparous women. Thus, it is suggested that having one or more children could be a strong protective factor against the BC of the luminal-A subtype. Several studies have explored the association of parity and BC molecular subtypes. A meta-analysis review by Lambertini et al. revealed that parity correlated with a significant reduction in risk of developing hormonal receptor-positive BC in parous women [36]. A similar finding was observed in a study from Northern China women aged below 41 years [14]. The mechanism on how parity decreases the risk of BC in luminal subtypes is postulated to be through modulation of circulating ER and PR hormones and acceleration of mammary gland tissues differentiation [37].

On the other hand, parity was reported to be associated with an increased risk for triple-negative subtypes in women irrespective of age at diagnosis [18]. Previous studies also showed that pregnancy has a cross effect for BC risk as a short-term effect. Pregnancy transiently increases the BC risk after birth due to stimulating the malignant cell transformation. However, as a long-term effect, it reduces the BC risk in later years due to inducing the differentiation of normal mammary stem cells [38]. More comprehensive case–control research is warranted in the SSA population to understand parity’s biological role in different BC molecular subtypes.

We demonstrated that the luminal-A subtype was more likely to be found in women with the first full-term pregnancy at 30 years or above compared to other subtypes. Our findings corroborates with previous studies that showed that having the first full-term pregnancy at 24 years or more increased the risk of hormonal receptor-positive BC [36]. A prior study in the Turkish population reported that women with the first full-term pregnancy after age 30 also had a significantly elevated risk of luminal BC [35]. Additionally, a recent study in women aged 40+ years of Northern China revealed similar results [14]. Another study in Latin American countries revealed that older age at the first full-term pregnancy was positively associated with the risk of BC overall [39].

Moreover, a recently published study observed that the first delivery at age ≥ 31 was associated with an increased risk of both IDC and ILC BC tumor subtypes in Iranian BC women [40]. The Breast Cancer Association Consortium (BCAC) reported that parity decreases the risk of BC by 16%, and each live birth reduces the risk of developing BC by 11%. Meanwhile, each 5-year increment in the age at first full-term pregnancy and the birth age was associated with a 7% increase in the risk of developing BC [41]. Our regression analysis showed that women with the first full-term pregnancy before 30 years were more likely to be diagnosed with luminal-B and triple-negative subtypes relative to the luminal-A subtype. Our observation partly agrees with Brouckaert et al. that reported a non-linear association of increasing age at first full-term pregnancy and triple-negative subtype [18].

Various studies show that breast-feeding for at least 6 months has protection against luminal subtypes [35] and triple-negative subtypes [42]. However, a recent case-only study from Kenya, an East Africa country, reported a lack of association of breast-feeding across all BC subtypes even after age stratification [43]. Our study showed that breast-feeding had a significant inversely association with luminal-A, luminal-B and triple-negative subtypes relative to women who never breast-fed. The majority of participants (57.8%) in this study cohort had breast-fed their babies at least 15 months. The effect of long-term breast-feeding has been reported to be overall protection against all BC subtypes [39]. Our data showed likely protection against luminal and triple-negative subtypes however there should be an accelerated effort to encourage Tanzanian women and other Africans, in general, to breast-feed their babies for a longer period as breast-feeding has positive outcomes for both the mother and the child. The mechanism of how breast-feeding protects against BC is well described in the literature. This protection works via hormonal mechanisms such as reducing estrogen levels in breast tissues, differentiation in mammary tissues, and apoptosis in progenitor cells [12, 44, 45].

Furthermore, we analyzed the combined effect of parity and breast-feeding in this study cohort. Our data revealed a non-significant decreased odds of developing luminal-A among women with at least one full-term pregnancy and breast-feeding. Contrary to our findings, a USA study reported that women under 50 years who had at least three full-term pregnancies and did not or breast-fed for less than 12 months had a twofold increased risk of triple-negative subtype [46]. We further recommend more extended period breast-feeding practice among parous African women. It has been shown that parity and breast-feeding are the modifiable risk factors, hence, considered indispensable tools toward BC prevention strategies.

Family history is a well-known risk factor of BC, but we could not find heterogeneity in BC or other types of cancers among the subtypes. Similar results were observed in a PreFace study that analyzed 3392 post-menopausal patients with hormone receptor-positive early BC in Germany [47] and a study of Turkish women [35]. However, contrary to our study, findings from South-East Asian ethnic groups reported that family history was only associated with triple-negative subtype after adjustment for grade or stage at BC diagnosis [48]. Additionally, our analysis found no heterogeneity of age at menarche among the four BC molecular subtypes. This was in agreement with the findings from the population-based case-case study in the USA [46] and divergent to a study in China that found women aged 13 years or below had increased odds of developing basal-like BC [47].

The strength of the current work has given the first impression of the prevalence of BC molecular subtypes and their association with reproductive factors in Tanzanian BC patients. However, our study has some limitations:

1. Our study did not consider survivorship bias across the four BC subtypes. It is known that triple-negative BC patients have the poorest survival when compared to other subtypes.
2. The case-only study design introduces some cautions in interpretation. Our sample size is relatively small (263 patients), and the design was a case-only study, not a case–control study that would have given the absolute risks of BC. In addition, BC molecular subtyping was based on IHC markers status since gene expression profiling is not available in clinical settings in most resource-poor countries like Tanzania. Still, it is powerful enough to give the first impression of BC etiology heterogeneity among the subtypes in a population of poor-resources.
3. IHC BC molecular classification did not include proliferative index Ki-67 and basal cytokeratin 5/6 (CK5/6).

These markers are not routinely performed in hospitals in resource-poor settings. Having data on these markers would help us further classify triple-negative cases into basal-like and normal-like subtypes. In addition, Ki-67 would help us classify exactly luminal-A and luminal-B subtypes.

## Conclusions

In conclusion, to the best of our knowledge, this is the first study from Tanzania to demonstrate the association between the BC molecular subtypes and the reproductive factors. Also, our data add to the existing knowledge of reproductive factors heterogeneity among BC molecular subtypes for Eastern African BC patients. The results showed that reproductive risk factors (menopausal status, parity, age at first full-term pregnancy, and breast-feeding) were associated with luminal BC in Tanzanian women. In general, older and post-menopausal women were more likely to be diagnosed with luminal-A subtype. In contrast, young and pre-menopausal women were more likely to be diagnosed with luminal-B subtype in Tanzanian BC patients.

Overall, our data concordance with recent publications, suggesting that breast-feeding may reduce the likelihood of developing luminal-A, luminal-B, and triple-negative subtypes. At the same time, the first full-term pregnancy after 30 years of age could increase the chance of developing luminal-A subtype, which is a highly prevalent subtype in Eastern African Tanzanian BC patients. Our study has presented the preliminary findings. Future work needs to be performed in large cohorts, a case–control study is required to confirm these associations of reproductive factors and BC subtypes in Africa.

## Supplementary Information

**Additional file 1**. The odds ratios and 95% confidence intervals of associated reproductive factors by BC subtypes (Table S1) and Distribution of BC molecular subtypes among selected African countries (Table S2).

## Data Availability

The datasets generated during and/or analyzed during the current study are available from the corresponding author on reasonable request.

## Abbreviations

SSA: Sub-Saharan Africa
BC: Breast cancer
IDC-NST: Invasive ductal carcinoma of no specific type
ILC: Infiltrating lobular carcinoma
IHC: Immunohistochemistry
BMI: Body mass index
EGFR: Epidermal growth factor receptor
ER: Estrogen receptor
HER2: Human epidermal growth factor receptor 2
HR: Hormonal receptor
HRT: Hormone replacement therapy
PR: Progesterone receptor
TNM: Tumor node metastasis
FFPE: Formalin-fixed paraffin-embedded
ORCI: Ocean Road Cancer Institute
MNH: Muhimbili National Hospital
KCMC: Kilimanjaro Christian Medical Center
BMC: Bugando Medical Center
NIMR: National Institute for Medical Research
MOEST: Tanzania Ministry of Education, Science and Technology
WHO: World Health Organization;
BCAC: Breast Cancer Association Consortium
CAP/ASCO: College of American Pathologist/American Society of Clinical Oncology
CI: Confidence interval
OR: Odds ratio
χ2: Chi-square
