# Supplementary material for: The distribution of reproductive risk factors disclosed the heterogeneity of receptor-defined breast cancer subtypes among Tanzanian women
Source: BMC Womens Health. 2021 Dec 20;21:423. doi: 10.1186/s12905-021-01536-6 (PMC8686374; doi:10.1186/s12905-021-01536-6)
Supplement: Supplementary file 1 — Additional file 1. The odds ratios and 95% confidence intervals of associated reproductive factors by BC subtypes (Table S1) and Distribution of BC molecular subtypes among selected African countries (Table S2). [file 12905_2021_1536_MOESM1_ESM.docx]

**Table S1:** The odds ratios and 95% confidence intervals of associated reproductive factors by breast cancer subtypes.

| Breast Cancer Subtypes | Luminal-A | Luminal-B | HER-2 enriched | Triple-negative |
| --- | --- | --- | --- | --- |
| Risk Factors | **OR (95% CI)**  **p-value** | **OR (95% CI)**  **p-value** | **OR (95% CI)**  **p-value** | **OR (95%CI)**  **p-value** |
| Age at diagnosis | | | | |
| <40 years old | 2.06 (1.25-3.39)  ***0.004*** | 1.73 (0.72-4.13)  0.214 | 0.73 (0.34-1.54)  0.412 | 1.16 (0.64-2.11)  0.617 |
| ≥40 years old |  |  |  |  |
| Menopausal status | | | | |
| Pre-menopause | 2.69 (1.16-4.47)  ***0.001*** | 3.89 (2.07-7.33)  ***0.001*** | 0.74 (0.35-1.58)  0.449 | 1.08 (0.59-1.97)  0.787 |
| Post-menopause |  |  |  |  |
| Age at first full-term pregnancy | | | | |
| <30 years old | 0.50 (0.26-0.95)  ***0.036*** | 1.73 (0.72-4.13)  0.214 | 0.96 (0.37-2.51)  0.943 | 1.82 (0.72-4.59)  0.200 |
| ≥30 years old |  |  |  |  |
| Parity | | | | |
| Nulliparous | 2.98 (1.23-7.18)  ***0.015*** | 0.64 (0.21-1.96)  0.440 | 0.44 (0.10-1.95)  0.284 | 0.69 (0.22-2.12)  0.527 |
| ≥1 Child |  |  |  |  |
| Breastfeeding | | | | |
| Yes | 1.98 (1.09-3.61)  ***0.025*** | 1.77 (0.93-3.36)  0.081 | 0.73 (0.28-1.88)  0.524 | 1.76 (0.91-3.38)  0.089 |
| No |  |  |  |  |

CI: Confidence Interval; OR: Odds Ratio; HER-2 Human Epidermal growth factor Receptor-2. The ORs and 95%CIs were found by comparing luminal-A to non-luminal-A; luminal-B to non-luminal-B; HER-2 enriched to non-HER-2 enriched and Triple-negative to non-Triple-negative cases. In all cases differences were considered significant at p<0.05.

| Region | Country | Luminal-A (%) | Luminal-B (%) | HER-2 Enriched (%) | Triple-Negative (%) | Studied Cases | Reference |
| --- | --- | --- | --- | --- | --- | --- | --- |
| **Northern Africa** | **Morocco** | 61.1 | 16.1 | 8.6 | 14.2 | 2260 | [[25](#_ENREF_25)] |
|  | **Egypt** | 56.7 | 16.7 | 11.7 | 15.0 | 60 | [[7](#_ENREF_7)] |
|  | **Algeria** | 41.86 | 15.09 | 13.46 | 29.58 | 676 | [[34](#_ENREF_34)] |
|  | **Tunisia** | 50.7 | 13.4 | 13.4 | 22.5 | 966 | [[49](#_ENREF_49)] |
|  | **Sudan** | 14.5 | 34.9 | 19.2 | 31.4 | 255 | [[50](#_ENREF_50)] |
|  |  | 35.0 | 12.8 | 16.7 | 35.5 | 437 | [[51](#_ENREF_51)] |
| **Western Africa** | **Nigeria** | 27.1 | 10.8 | 5.5 | 56.6 | 417 | [[52](#_ENREF_52)] |
|  |  | 28.8 | 6.8 | 17.8 | 46.6 | 118 | [[53](#_ENREF_53)] |
|  |  | 52.4 | 12.9 | 8.2 | 26.5 | 147 | [[54](#_ENREF_54)] |
|  | **Ivory Coast** | 46.4 | 11.8 | 6.4 | 35.4 | 577 | [[55](#_ENREF_55)] |
|  |  | 51.6 | 10.1 | 6.3 | 32.1 | 287 | [[31](#_ENREF_31)] |
|  | **Ghana** | 25.6 | 12.2 | 12.8 | 49.4 | 165 | [[11](#_ENREF_11)] |
|  | **Guinea** | 35.7 | 14.3 | 23.2 | 26.8 | 56 | [[56](#_ENREF_56)] |
|  | **Mali** | 31.1 | 14.2 | 4.7 | 50.0 | 106 | [[57](#_ENREF_57)] |
| **Central Africa** | **Congo DR** | 62.1 | 14.9 | 8.0 | 14.9 | 87 | [[58](#_ENREF_58)] |
|  | **Angola** | 25.7 | 27.2 | 15.7 | 31.4 | 140 | [[59](#_ENREF_59)] |
| **Southern Africa** | **South Africa** | 53.7 | 14.6 | 11.4 | 20.4 | 1027 | [[60](#_ENREF_60)] |
|  | **Botswana** | 62.6 | 9 | 7.1 | 21.3 | 211 | [[61](#_ENREF_61)] |
| **Eastern Africa** | **Kenya** | 14.3 | 40.8 | 8.2 | 36.7 | 49 | [[29](#_ENREF_29)] |
|  | **Uganda** | 38 | 5 | 22 | 34 | 226 | [[62](#_ENREF_62)] |
|  | **Ethiopia** | 40.2 | 26.8 | 9.8 | 23.2 | 112 | [[10](#_ENREF_63)] |
|  | **Eritrea** | 36.2 | 12.1 | 17.2 | 34.5 | 116 | [[51](#_ENREF_51)] |
|  | **Tanzania** | 44.5 | 22.4 | 11 | 22.1 | 263 | Present study |

**Table S2:** Distribution of BC molecular subtypes among selected African countries
